# Supplementary material for: Thermodynamic Characterization of Rhamnolipid, Triton X-165 and Ethanol as well as Their Mixture Behaviour at the Water-Air Interface
Source: Molecules. 2023 Jun 25;28(13):4987. doi: 10.3390/molecules28134987 (PMC10343227; doi:10.3390/molecules28134987)
Supplement: Supplementary file 1 [file molecules-28-04987-s001.zip › molecules-2448077-supplementary.pdf]

Supplementary Information

THERMODYNAMIC CHARACTERIZATION OF RHAMNOLIPID, TRITON X-165 AND ETHANOL AS WELL AS THEIR MIXTURE BEHAVIOUR AT THE WATER-AIR INTERFACE

Anna Zdziennicka[1], Maria Luisa Gonzalez-Martin[2,3]*, Edyta Rekiel[1], Katarzyna Szymczyk[1], Wojciech Zdziennicki[4] and Bronisław Jańczuk[1]

[1]Department of Interfacial Phenomena, Institute of Chemical Sciences, Faculty of Chemistry, Maria Curie-Skłodowska University in Lublin, Maria Curie-Skłodowska Sq. 3, 20-031 Lublin, Poland;

[2]Department of Applied Physics, Extremadura University, Avda. de Elvas, s/n, 06006 Badajoz, Spain;

[3]Networking Research Center on Bioengineering, Biomaterials and Nanomedicine (CIBER-BBN), 06006 Badajoz, Spain;
mlglez@unex.es (M.L.G.-M.)
[4]University Clinical Hospital in Poznań, Przybyszewskiego 49, 60-355 Poznań, Poland;

Table S1. The constant of adsorption (a) from the Szyszkowski and linear Langmuir equations as well as Gibbs standard free energy of adsorption (ΔG^0_{ads}).

Individual compounds					
**ET**		**RL**		**TX165**	
a	ΔG^0_{ads} (mJ/mol)	a	ΔG^0_{ads} (mJ/mol)	a	ΔG^0_{ads} (mJ/mol)
0.529777[a]	-11.33[a]	3.60602E-07[a]	-45.92[a]	3.51729E-07[a]	-45.98[a]
0.53106[c]	-11.32[c]	5.73E-7[b]	-44.79[b]	4.74849E-7[b]	-45.25[b]
		1.46471E-6[c]	-42.51[c]	9.44033E-7[c]	-43.58[c]

Binary mixtures

ET + RL

C_{RL} (mg/dm³)	ET		RL	
	a	ΔG^0_{ads} (mJ/mol)	a	ΔG^0_{ads} (mJ/mol)
0.01	0.706247[a]<br>0.62219[c]	-10.63[a]<br>-10.84[c]	1.55521E-6[c]	-42.26[c]
0.5	1.090464[a]<br>0.7695[c]	-9.57c<br>-10.08c	3.0569E-7[c]	-45.98[c]
5	1.258248[a]<br>0.5144[c]	-9.22[a]<br>-10.74[c]	1.03389E-5[c]	-37.09[c]
20	0.982206[a]<br>0.3951[c]	-9.82[a]<br>-10.90[c]	3.08236E-5[c]	-33.94[c]

TX165 + RL

C_{RL} (mg/dm³)	TX165		RL	
	a	ΔG^0_{ads} (mJ/mol)	a	ΔG^0_{ads} (mJ/mol)
0.01	7.01489E-08[a]<br>3.45E-7[b]<br>2.42958E-7[c]	-49.91[a]<br>-46.03[b]<br>-46.88[c]	1.93313E-7[c]	-47.44[c]
0.5	1.31912E-06[a]<br>4.35E-6[b]<br>8.45E-07[c]	-42.76[a]<br>-39.85[b]<br>-43.85[c]	2.09135E-6[c]	-41.64[c]
5	2.04808E-06[a]<br>1.90E-06[c]	-41.69[a]<br>-41.88[c]	1.25667E-5[c]	-37.27[c]
20	1.43641E-06[a]<br>1.81E-06[c]	-42.55[a]<br>-42.00[c]	4.81185E-5[c]	-34.00[c]

TX165 + ET

C_{ET} (mole/dm³)	TX165		ET	
	a	ΔG^0_{ads} (mJ/mol)	a	ΔG^0_{ads} (mJ/mol)
1.07	6.27268E-07[a]<br>2.67E-6[b]<br>7.41E-07[c]	-44.57[a]<br>-40.95[b]<br>-44.07[c]	1.8763[c]	-8.15[c]
3.74	6.79548E-07[a]<br>8.76E-6[b]<br>7.90E-07[c]	-44.38[a]<br>-37.81[b]<br>-43.67[c]	4.59999[c]	-5.72[c]
6.69	7.5454E-07[a]<br>7.81E-07[c]	-44.12[a]<br>-43.38[c]	7.6391[c]	-4.17[c]
10.27	8.16562E-07[a]<br>7.79036E-7[c]	-43.93[a]<br>-42.90[c]	11.42298[c]	-2.70[c]

Ternary mixtures ET + RL + TX165						
ET			RL		TX165	
C_{RL} = 0.01 (mg/dm[3])						
C_{ET} (mole/dm[3])	a	ΔG^0_{ads} (mJ/mol)	a	ΔG^0_{ads} (mJ/mol)	a	ΔG^0_{ads} (mJ/mol)
1.07	1.91901[c]	-8.10[c]	1.35E-6[c]	-42.71[c]	8.87628E-07[a]<br>1.67E-6[b]<br>8.15847E-7[c]	-43.73[a]<br>-42.09[b]<br>-43.93[c]
3.74	4.68117[c]	-5.68[c]	1.37E-6[c]	-42.66[c]	8.01378E-07[a]<br>5.43E-6[b]<br>8.2334E-7[c]	-43.98[a]<br>-38.97[c]<br>-43.91[c]
6.69	7.72081[c]	-4.14[c]	1.35E-6[c]	-42.70[c]	9.15922E-07[a]<br>8.04582E-7[c]	-43.65[a]<br>-43.97[c]
10.27	11.51866[c]	-2.68[c]	1.35E-6[c]	-42.71[c]	8.66665E-07[a]<br>8.02366E-7[c]	-43.78[a]<br>-43.97[c]
C_{RL} = 0.5 (mg/dm[3])						
1.07	2.20037[c]	-7.76[c]	2.71E-6[c]	-41.01[c]	1.29553E-06[a]<br>3.52E-6[b]<br>9.77306E-7[c]	-42.81[a]<br>-42.09[b]<br>-43.49[c]
3.74	5.23458[c]	-5.40[c]	2.85E-6[c]	-40.89[c]	1.51102E-06[a]<br>7.98E-6[b]<br>1.03502E-6[c]	-42.43[a]<br>-38.03[b]<br>-43.35[c]
6.69	8.16887[c]	-4.01[c]	2.64E-6[c]	-41.07[c]	1.29075E-06[a]<br>9.34968E-7[c]	-42.81[a]<br>-43.60[c]
10.27	12.02144[c]	-2.58[c]	2.59E-6[c]	-41.12[c]	1.10939E-06[a]<br>9.14786E-7[c]	-43.18[a]<br>-43.65[c]
C_{RL} = 5 (mg/dm[3])						
1.07	2.87718[c]	-7.11[c]	1.52E-5[c]	-36.81[c]	2.61519E-06[a]<br>5.67E-6[b]<br>1.36628E-6[c]	-41.09[a]<br>-39.11[b]<br>-42.68[c]
3.74	6.46327[c]	-4.89[c]	1.57E-5[c]	-36.73[c]	2.45759E-06[a]<br>5.67E-6[b]<br>1.46688E-6[c]	-41.25[a]<br>-38.87[b]<br>-42.50[c]
6.69	9.35242[c]	-3.68[c]	1.46E-5[c]	-36.90[c]	1.88616E-06[a]<br>1.24779E-6[c]	-41.89[a]<br>-42.90[c]
10.27	13.70777[c]	-2.26[c]	1.46E-5[c]	-36.90[c]	1.85605E-06[a]<br>1.24712E-6[c]	-41.93[a]<br>-42.90[c]
C_{RL} = 20 (mg/dm[3])						
1.07	2.4993[c]	-7.45[c]	5.07E-5[c]	-33.87[c]	1.70465E-06[a]<br>1.14315E-6[c]	-42.14[a]<br>-43.11[c]
3.74	6.32273[c]	-4.94[c]	5.34E-5[c]	-33.75[c]	2.1713E-06[a]<br>1.41522E-6[c]	-41.55[a]<br>-42.59[c]
6.69	10.23963[c]	-3.46[c]	5.4E-5[c]	-33.72[c]	2.28014E-06[a]<br>1.46873E-6[c]	-41.43[a]<br>-43.11[c]
10.27	14.92871[c]	-2.05[c]	5.4E-5[c]	-33.72[c]	2.33992E-06[a]<br>1.46781E-6[c]	-41.37[a]<br>-42.59[c]

[a] – the values obtained based on the linear Langmuir equation

[b] – the values obtained based on the Szyszkowski equation, [c] – the values obtained based on $\frac{C}{X^S}$

a)

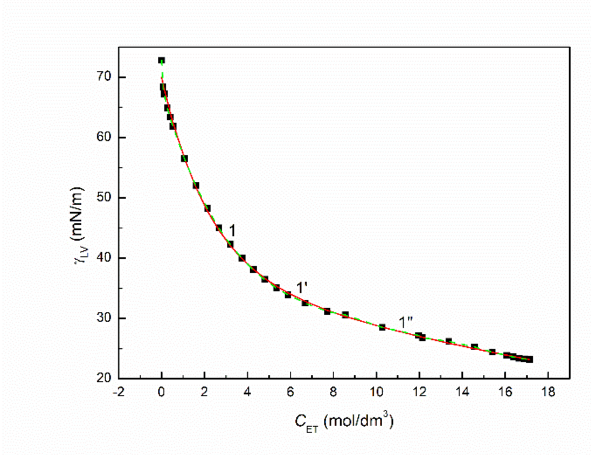

b)

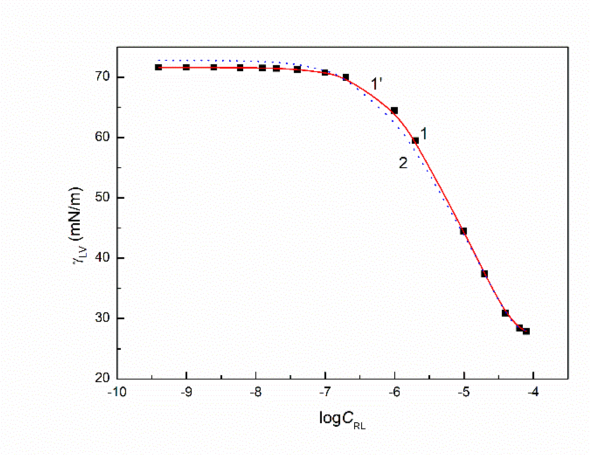

c)

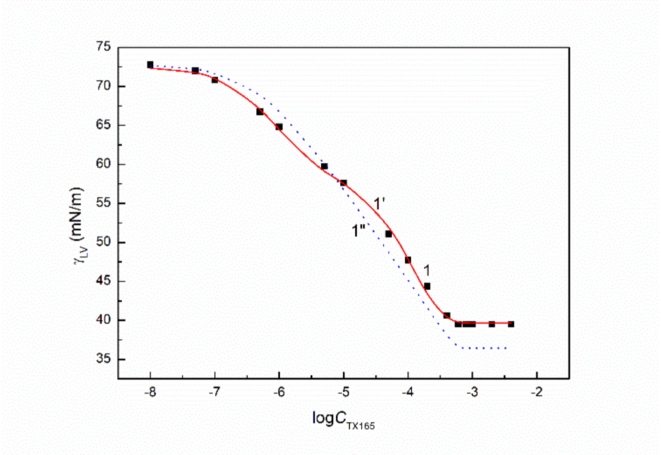

Figure S1. A plot of the surface tension (γ_{LV}) of the aqueous solution of ET as function of its concentration (C_{ET}) (a), surface tension of the aqueous solution of RL (b) and TX165 (c) as function of the logarithm of its concentration (log$C$). Points 1 correspond to the measured values, curves 1′ and 1″ correspond to the values calculated from Equations (1) and (3), curve 2 correspond to values calculated from Eq. (2), respectively.

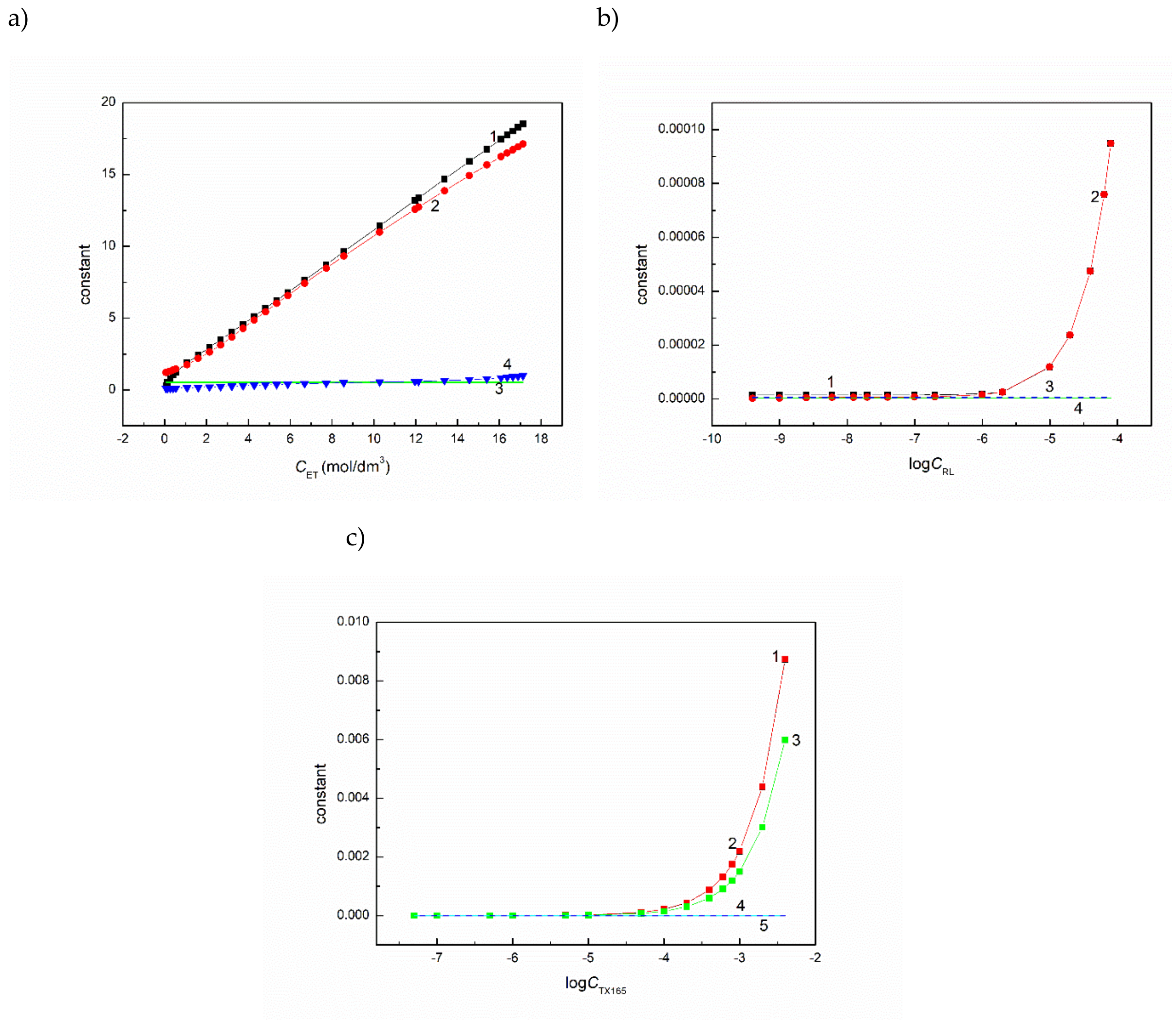

Figure S2. A plot of $\frac{C}{X^S}$ (curves 1 and 2), a (curve 3) and $\frac{a_{ET}}{X^S}$ (curve 4) for ET vs. its concentration (C_{ET}) (a) (curve 1 and 2 obtained using X^S calculated based on the Frumkin and our Equations, respectively, curve 3 correspond to the values calculated from Equation (2) and curve 4 to the obtained based on ET activity (a_{ET}) as well plot of $\frac{C}{X^S}$ (curves 1 and 2), a (curves 3 and 4) for RL (b) and TX165 (c) vs. the logarithm of its concentration ($\log C$) (curves 1 – 3 the same as in Figure S2a and curves 4 and 5 obtained from the Szyszkowski equation (Equation (2)).

a)

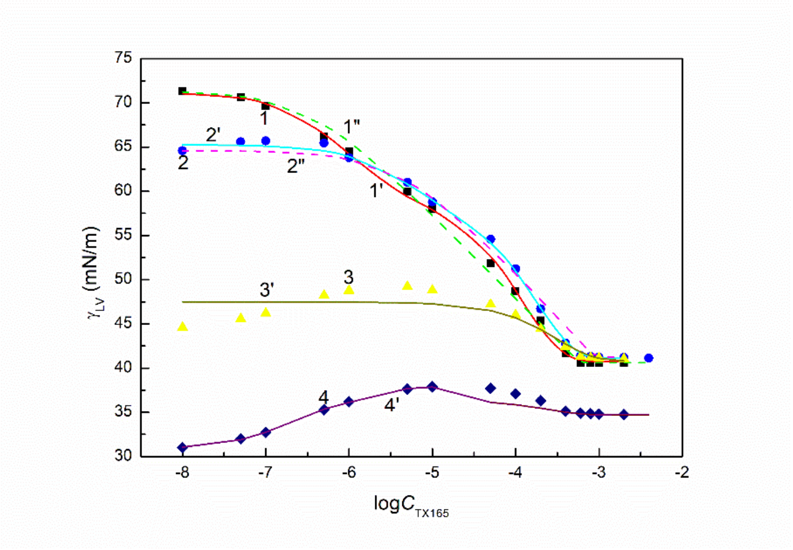

b)

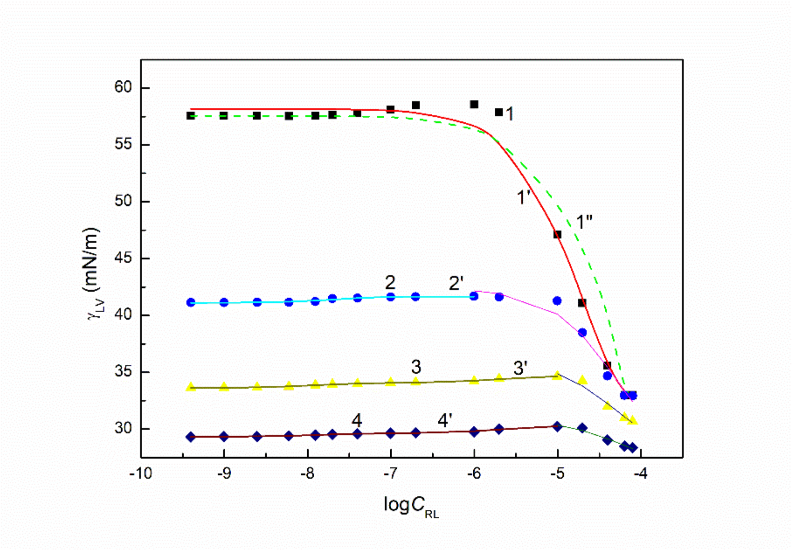

Figure S3. A plot of the surface tension (γ_{LV}) of the aqueous solution of the RL + TX165 mixture (a) at the constant RL concentration equal to 0.01 mg/dm^3 (points 1, curves 1′ and 1″), 0.5 mg/dm^3 (points 2, curves 2′ and 2″), 5 mg/dm^3 (points 3 and curve 3′) and 20 mg/dm^3 (points 4 and curve 4′) vs. the logarithm of TX165 concentration (logC_{TX165}) as well as plot of the surface tension of the aqueous solution of the RL + ET mixture (b) at the constant ET concentration equal to 1.07 mol/dm^3 (points 1, curves 1′ and 1″), 3.74 mol/dm^3 (points 2 and curve 2′), 6.69 mol/dm^3 (points 3 and curve 3′) and 10.27 mol/dm^3 (points 4 and curve 4′) vs. the logarithm of the RL concentration (logC_{RL}). Points 1 – 4 correspond to the measured values, curves 1′ – 4′ correspond to the values calculated from Equation (1) and curves 1″ and 2″ correspond to the values calculated from the Szyszkowski eqution (Equation (2)).

a)

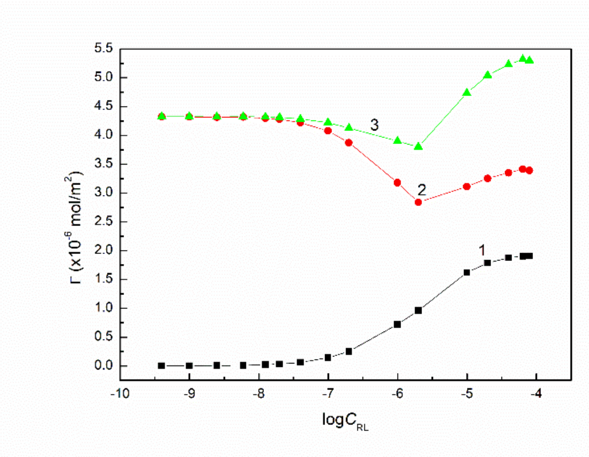

b)

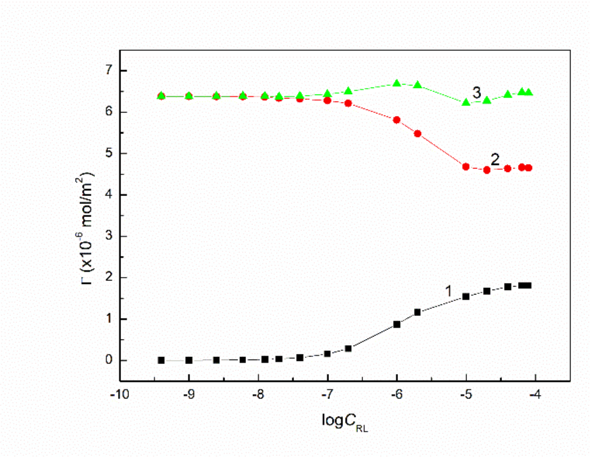

c)

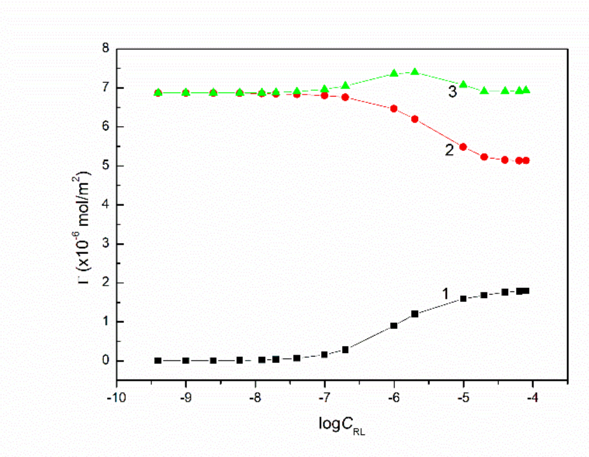

d)

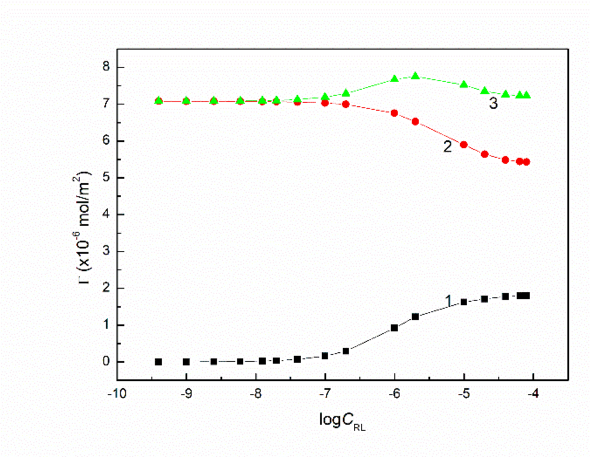

Figure S4. A plot of the Frumkin concentration at the solution-air interface (Γ) of RL (curve 1), ET (curve 2) and their sum (curve 3) at the constant ET concentration equal to 1.07 mol/dm³ (a), 3.74 mol/dm³ (b), 6.69 mol/dm³ (c) and 10.27 mol/dm³ (d) vs. the logarithm of the RL concentration ($\log C_{RL}$).

a)

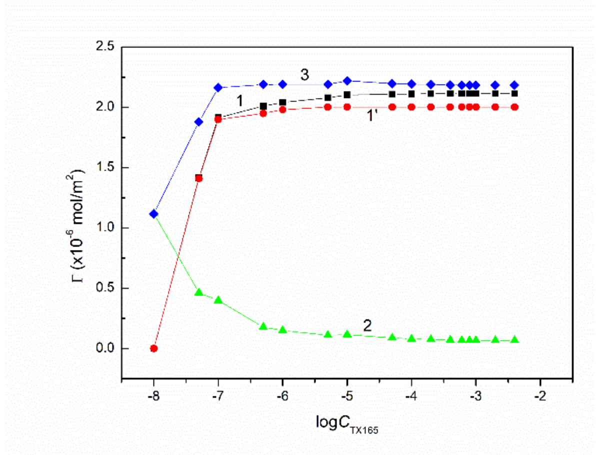

b)

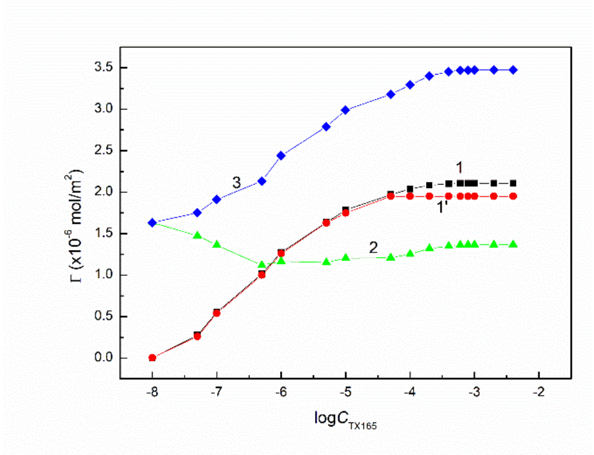

c)

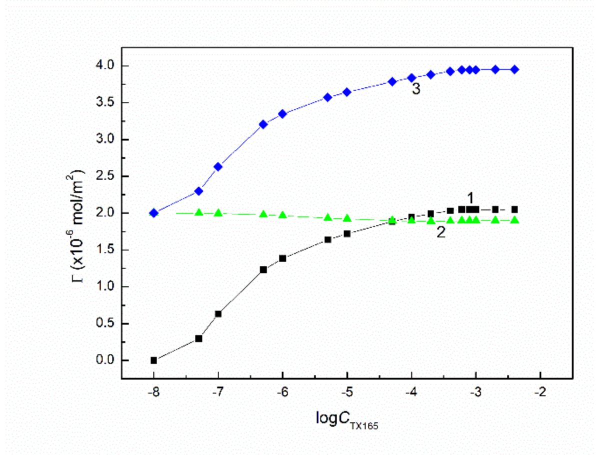

d)

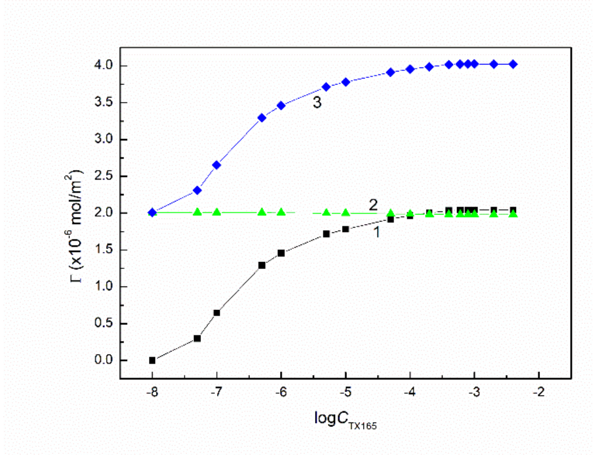

Figure S5. A plot of the Gibbs concentration (Γ) of TX165 (curve 1′) as well as the Frumkin concentration (Γ) of TX165 (curve 1), RL (curve 2) and their sum (curve 3) at the constant RL concentration equal to 0.01 mg/dm³ (a), 0.5 mg/dm³ (b), 5 mg/dm³ (c) and 20 mg/dm³ (d) vs. the logarithm of the TX165 concentration (C_{TX165}).

a)

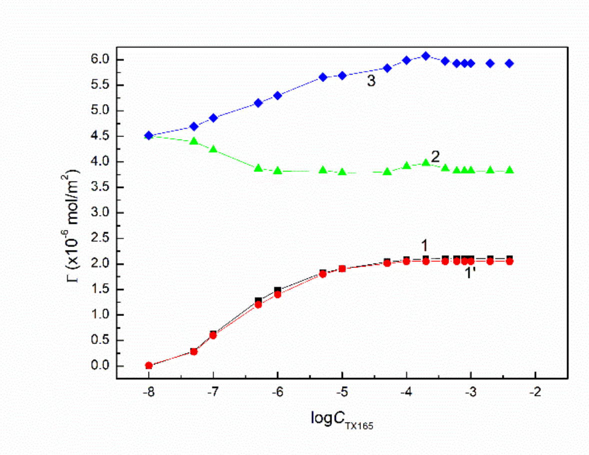

b)

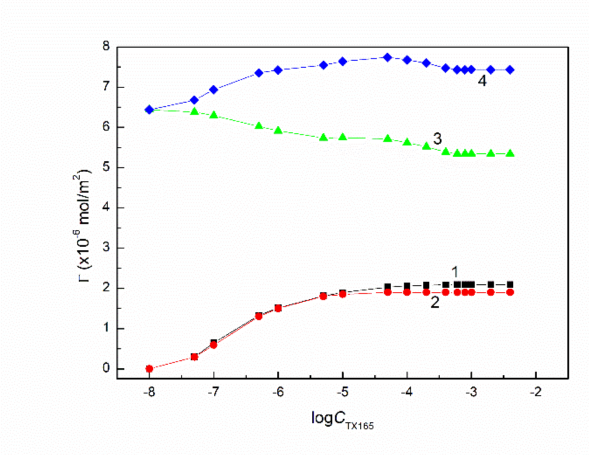

c)

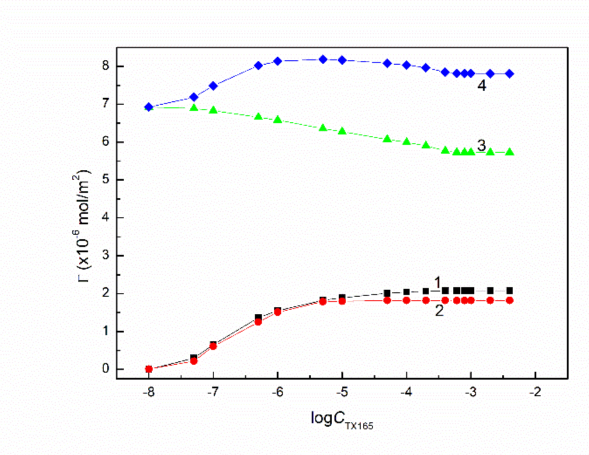

d)

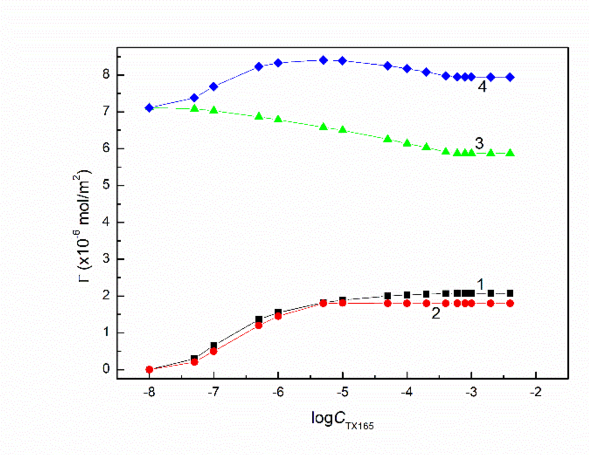

Figure S6. A plot of the Gibbs concentration (Γ) of TX165 (curve 1′) as well as the Frumkin concentration (Γ) of TX165 (curve 1), ET (curve 2) and their sum (curve 3) at the constant ET concentration equal 1.07 mol/dm³ (a), 3.74 mol/dm³ (b), 6.69 mol/dm³ (c) and 10.27 mol/dm³ (d) vs. the logarithm of the TX165 concentration (C_{TX165}).

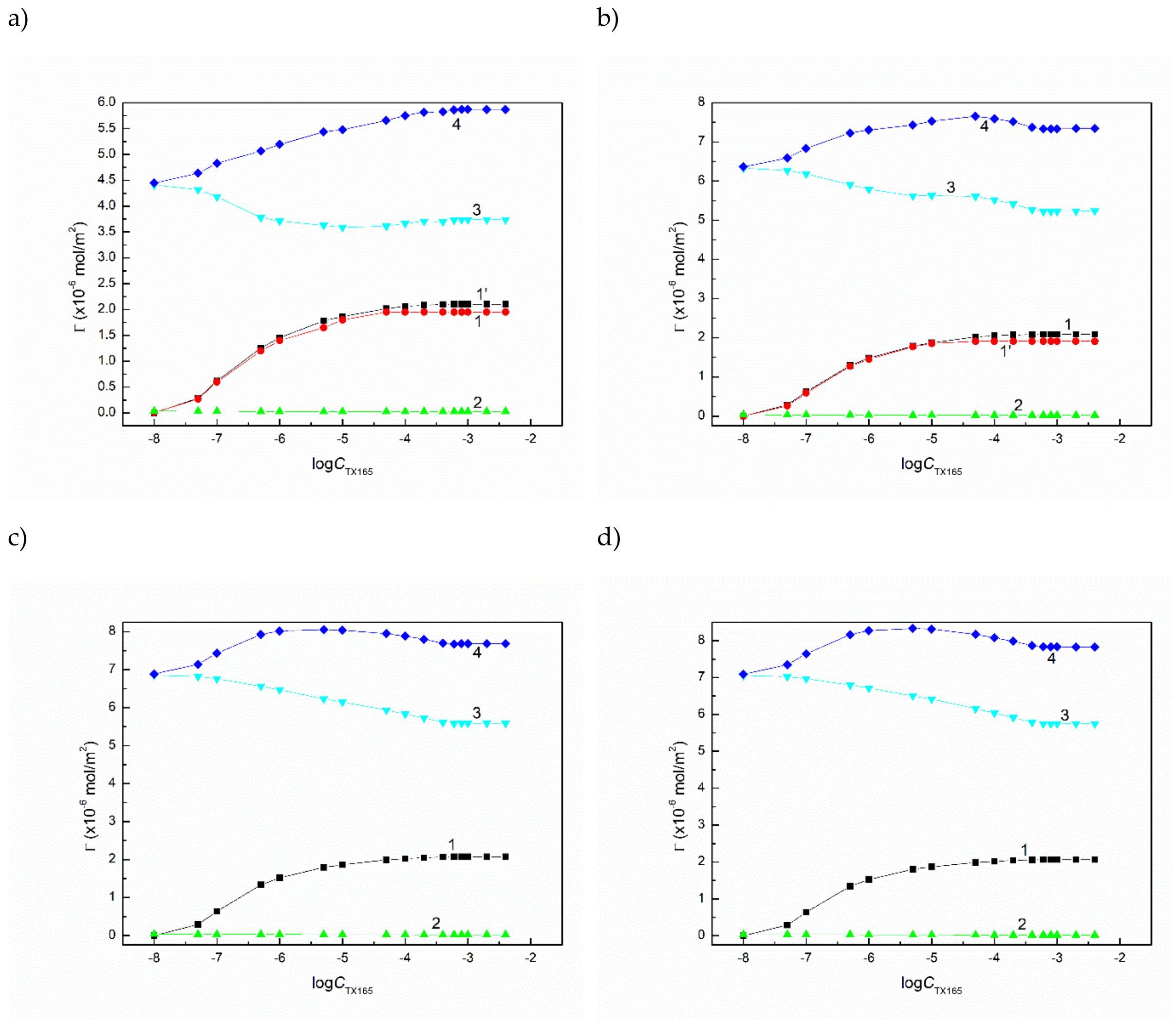

Figure S7. A plot of the Gibbs concentration (Γ) of TX165 (curve 1′) as well as the Frumkin concentration (Γ) of TX165 (curve 1), RL (curve 2), ET (curve 3) and their sum (curve 4) vs. the logarithm of the TX165 concentration (C_{TX165}) at the constant RL concentration equal to 0.01 mg/dm^3 and constant ET concentration equal to 1.07 mol/dm^3 (a), 3.74 mol/dm^3 (b), 6.69 mol/dm^3 (c) and 10.27 mol/dm^3 (d).

a) b) c) d)

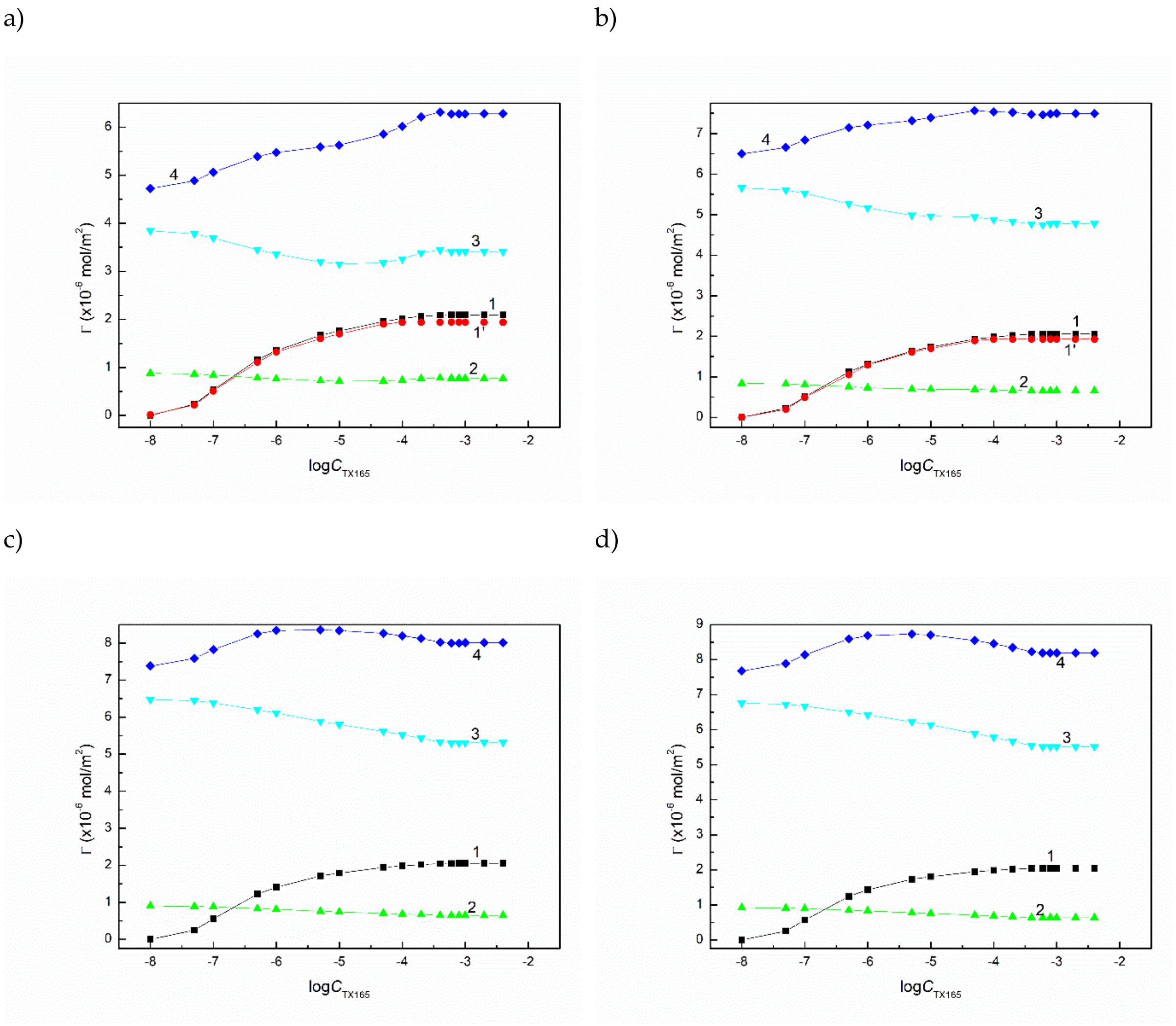

Figure S8. A plot of the Gibbs concentration (Γ) of TX165 (curve 1′) as well as the Frumkin concentration (Γ) of TX165 (curve 1), RL (curve 2), ET (curve 3) and their sum (curve 4) vs. the logarithm of the TX165 concentration (C_{TX165}) at the constant RL concentration equal to 0.5 mg/dm³ and constant ET concentration equal to 1.07 mol/dm³ (a), 3.74 mol/dm³ (b), 6.69 mol/dm³ (c) and 10.27 mol/dm³ (d).

a)

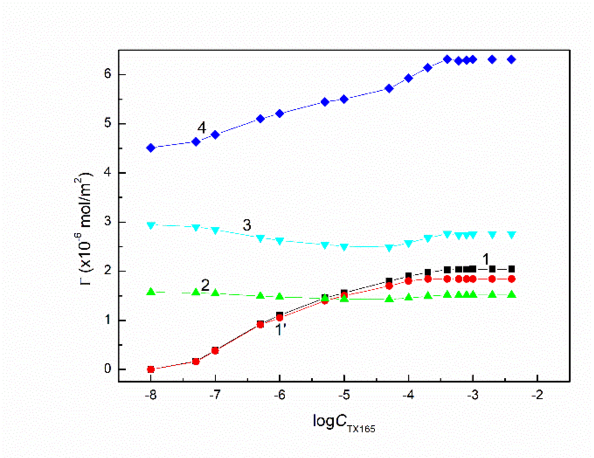

b)

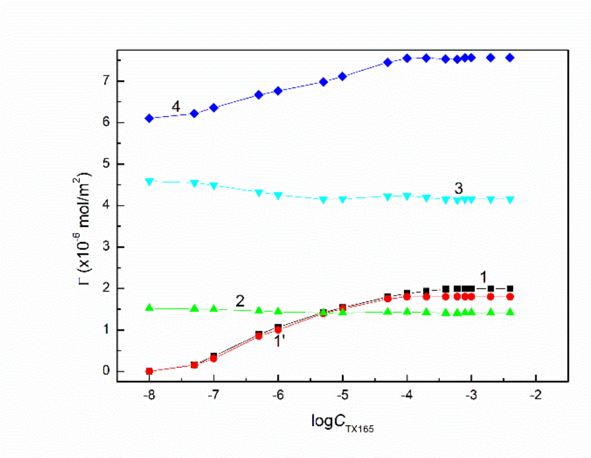

c)

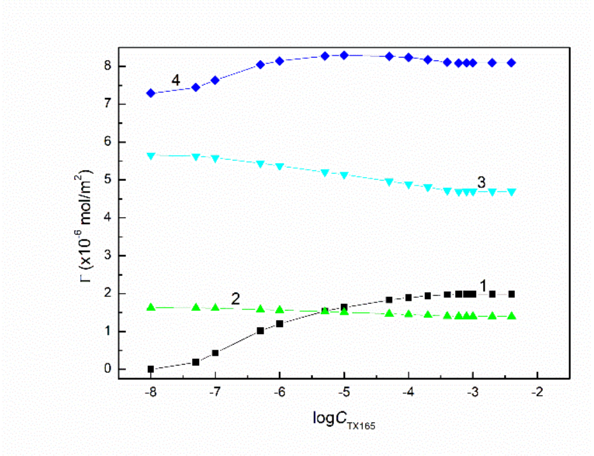

d)

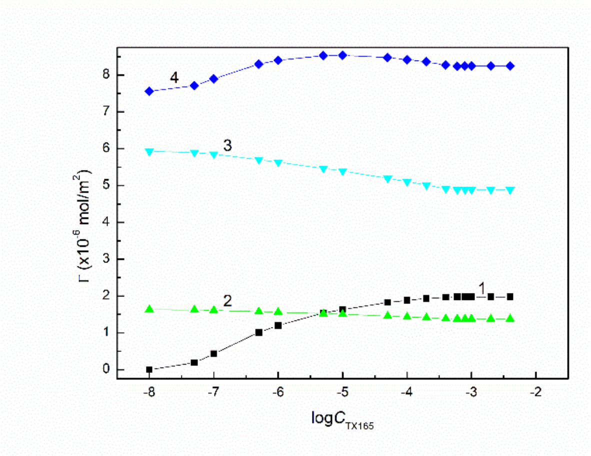

Figure S9. A plot of the Gibbs concentration (Γ) of TX165 (curve 1′) as well as the Frumkin concentration (Γ) of TX165 (curve 1), RL (curve 2), ET (curve 3) and their sum (curve 4) vs. the logarithm of the TX165 concentration (C_{TX165}) at the constant RL concentration equal to 5 mg/dm³ and constant ET concentration equal to 1.07 mol/dm³ (a), 3.74 mol/dm³ (b), 6.69 mol/dm³ (c) and 10.27 mol/dm³ (d).

a)

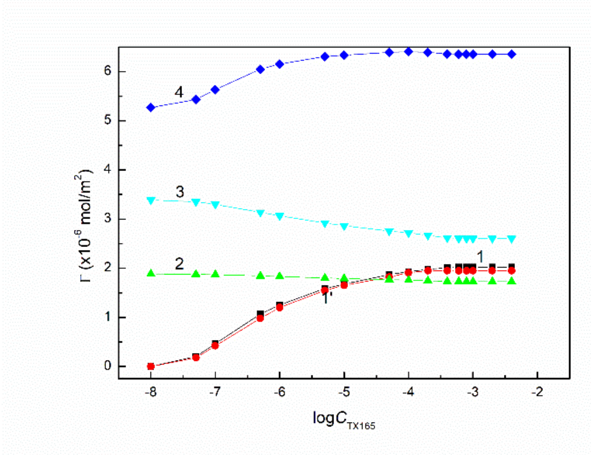

b)

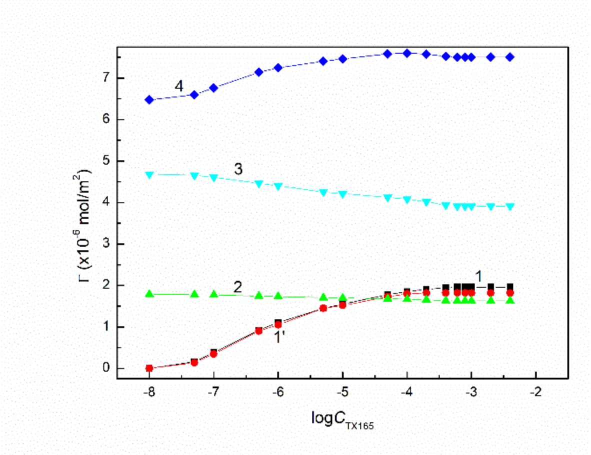

c)

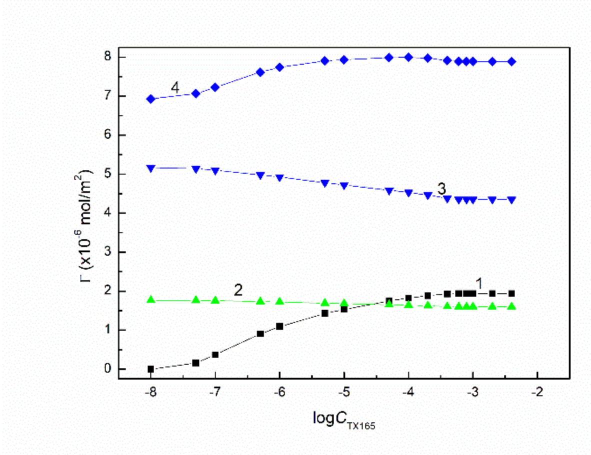

d)

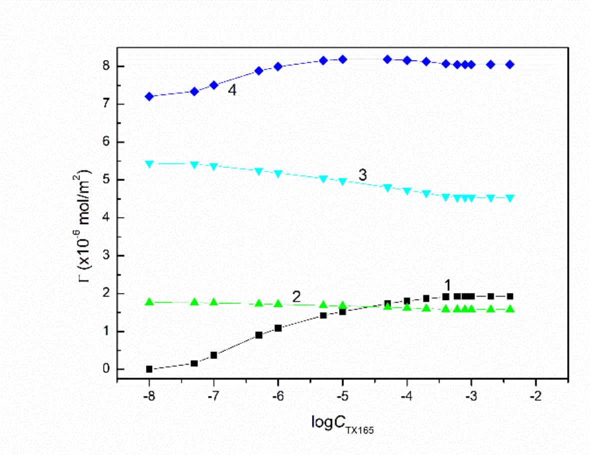

Figure S10. A plot of the Gibbs concentration (Γ) of TX165 (curve 1′) as well as the Frumkin concentration (Γ) of TX165 (curve 1), RL (curve 2), ET (curve 3) and their sum (curve 4) vs. the logarithm of the TX165 concentration (C_{TX165}) at the constant RL concentration equal to 20 mg/dm^3 and constant ET concentration equal to 1.07 mol/dm^3 (a), 3.74 mol/dm^3 (b), 6.69 mol/dm^3 (c) and 10.27 mol/dm^3 (d).

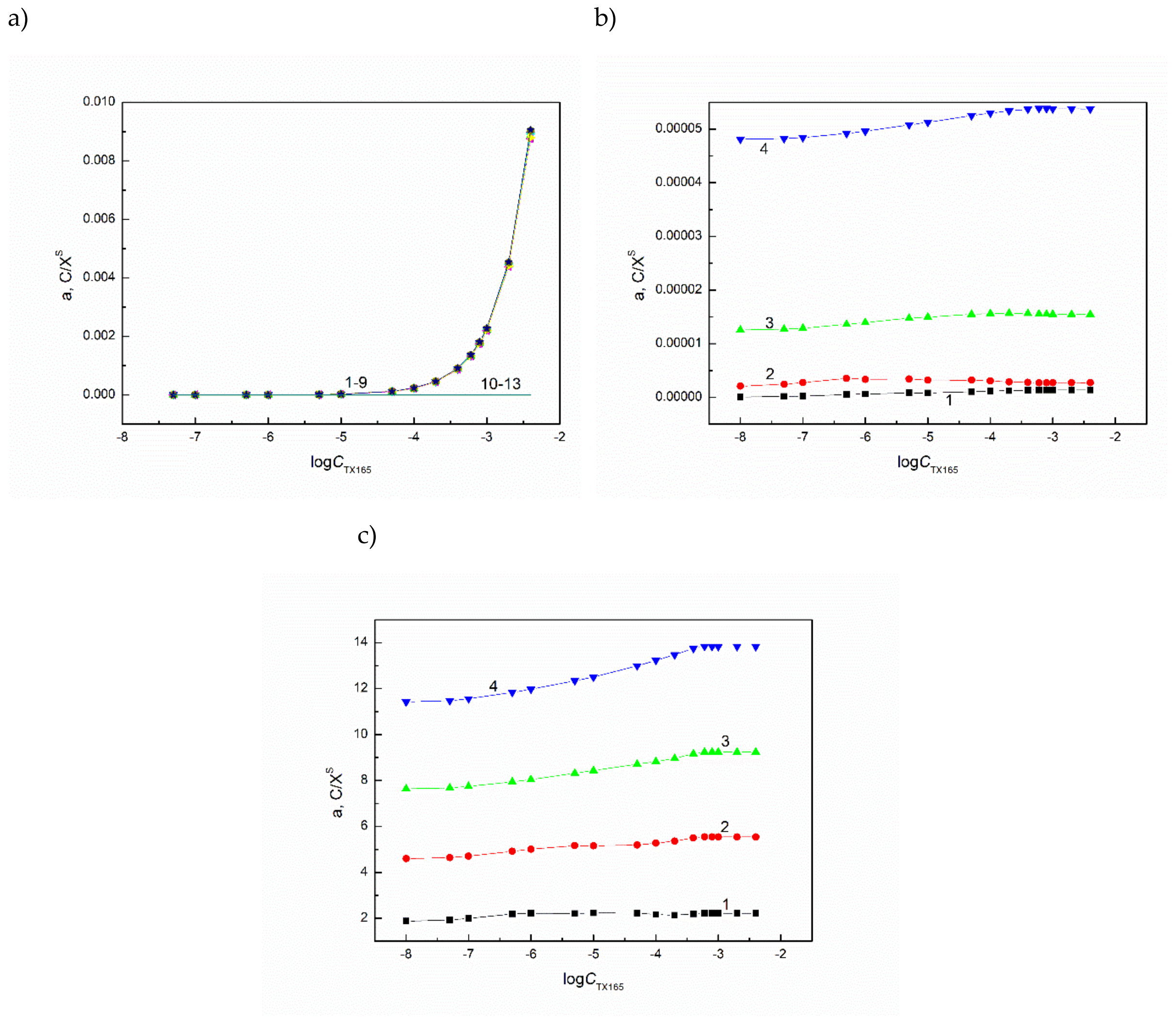

Figure S11. A plot of $\frac{C}{X^S}$ (curves 1 – 9) and a (curves 10 – 13) for TX165 (a), RL (b) and ET (c) vs. the logarithm of the TX165 concentration (C_{TX165}). The values of $\frac{C}{X^S}$ were obtained based on the Figures S5 and S6. The values of a were calculated based on the Szyszkowski (curves 10 and 11) and the linear Langmuir equations (curves 12 and 13) for the constant RL concentration equal to 0.01 mg/dm^3 and ET concentration 1.07 mol/dm^3.

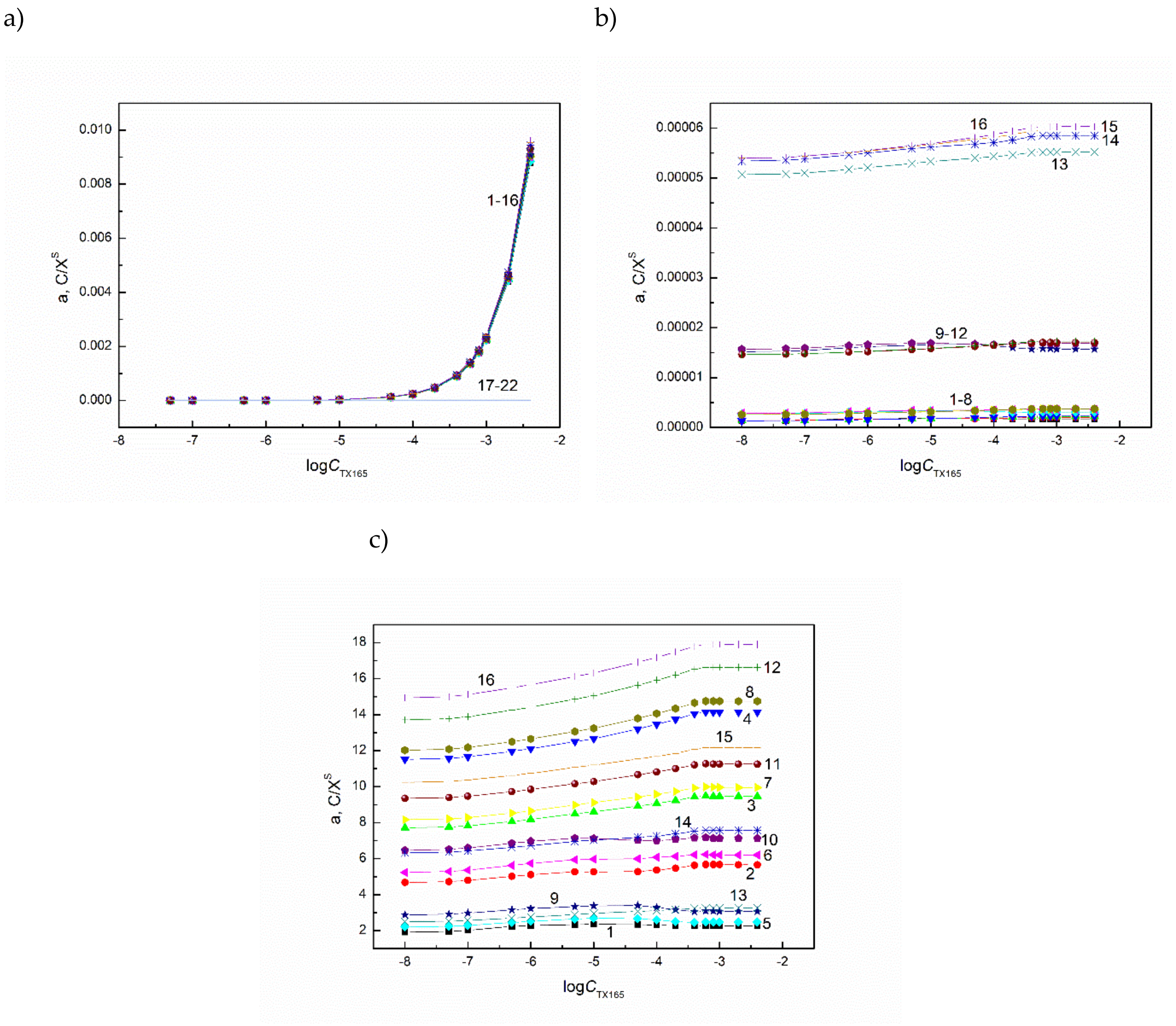

Figure S12. A plot of $\frac{C}{X^S}$ (curves 1 – 16) and a (curves 17 – 22) for TX165 (a), RL (b) and ET (c) vs. the logarithm of theTX165 concentration (C_{TX165}). The values of $\frac{C}{X^S}$were obtained based on the Figures S7 – S10. The values a were calculated based on the Szyszkowski (curves 17 - 19) and linear Langmuir equations (curves 20 - 22) for the constant RL concentration 0.01 mg/dm^3 and ET concentration 1.07 mol/dm^3. Curves 1 – 4 correspond to the constant RL concentration equal 0.01 mg/dm^3, curves 5 – 8 mg/dm^3 to 0.5 mg/dm^3, curves 9 – 12 mg/dm^3 to 5 and curves 13 – 16 to the RL concentration equal to 20 mg/dm^3.

a)

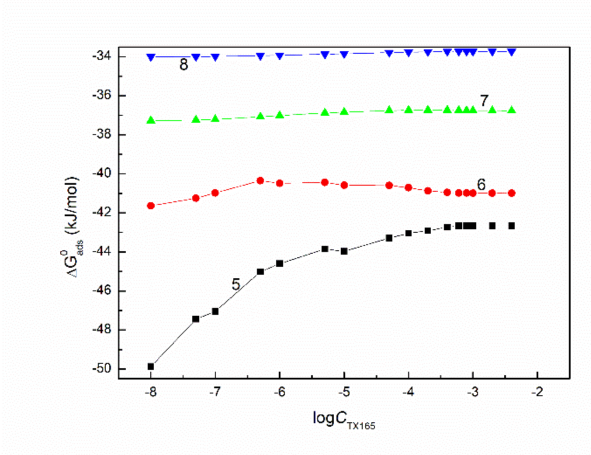

b)

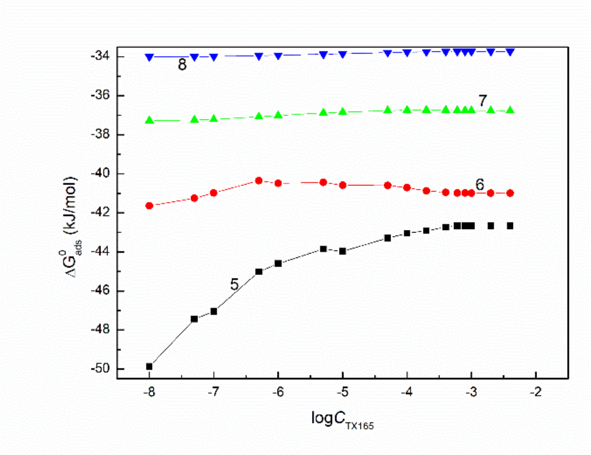

c)

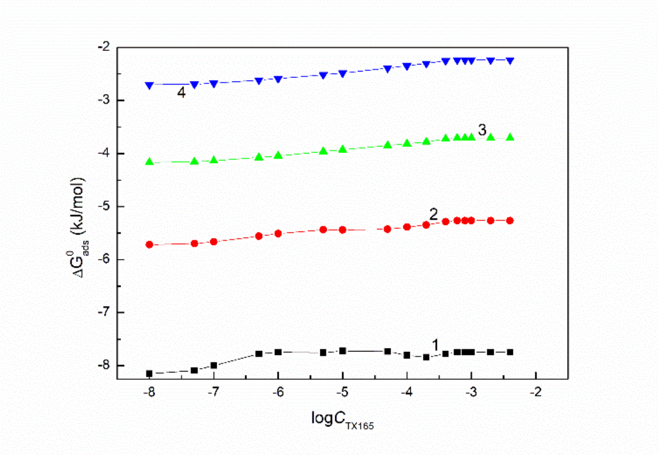

Figure S13. A plot of the standard Gibbs free energy of adsorption (ΔG^0_{ads}) calculated from Equation (19) for TX165 (a), RL (b) and ET (c) vs. the logarithm of the TX165 concentration (C_{TX165}). Curves 1 – 4 correspond to the constant ET concentration equal 1.07, 3.74, 6.69 and 10.27 mol/dm^3, curves 5 – 8 correspond to the constant RL concentration equal 0.01, 0.5, 5 and 20 mg/dm^3.

a)

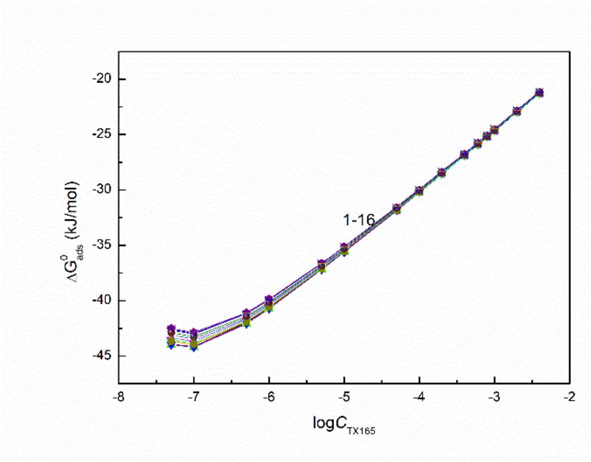

b)

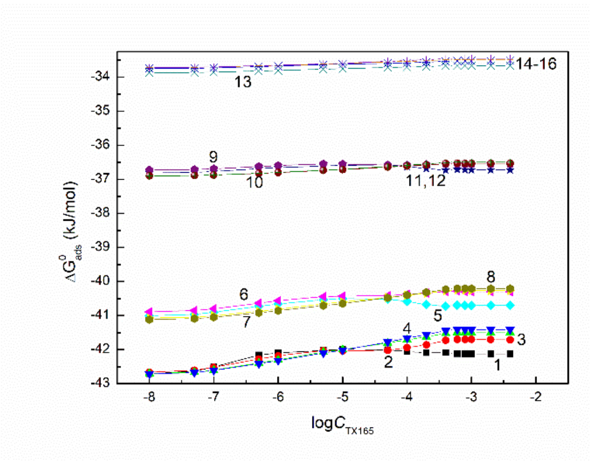

c)

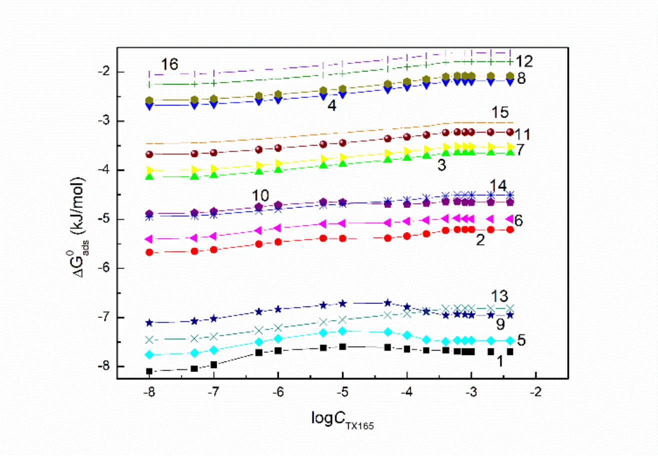

Figure S14. A plot of the standard Gibbs free energy of adsorption (ΔG^0_{ads}) calculated from Equation (19) for TX165 (a), RL (b) and ET (c) vs. the logarithm of the TX165 concentration (C_{TX165}). Curves 1 – 4 correspond to the constant ET concentration equal 1.07, 3.74, 6.69 and 10.27 mol/dm^3. Curves 1 – 4 correspond to the constant RL concentration equal 0.01 mg/dm^3, curves 5 – 8 mg/dm^3 to 0.5 mg/dm^3, curves 9 – 12 mg/dm^3 to 5 and curves 13 – 16 to the RL concentration equal to 20 mg/dm^3. Curves 1, 5, 9 and 13 correspond to the constant ET concentration equal to 1.07 mol/dm^3, curves 2, 6, 10 and 14 correspond to the constant ET concentration equal to 3,74 mol/dm^3, curves 3, 7, 11 and 15 correspond to the constant ET concentration equal to 6,69 mol/dm^3, curves 4, 8, 12 and 16 correspond to the constant ET concentration equal to 10.27 mol/dm^3,
